# Successful surgical treatment of an intraoperatively ruptured lung abscess rupture using free pericardial fat implantation: a case report

**DOI:** 10.1186/s40792-024-01814-z

**Published:** 2024-01-11

**Authors:** Yoshihito Iijima, Takaki Mizoguchi, Masahito Ishikawa, Shun Iwai, Nozomu Motono, Hidetaka Uramoto

**Affiliations:** Department of Thoracic Surgery, Kanazawa Medical University, 1-1 Daigaku, Uchinada-Machi, Kahoku-Gun, Ishikawa, 920-0293 Japan

**Keywords:** Lung abscess rupture, Pyothorax, Free pericardial fat, Surgery, Implantation

## Abstract

**Background:**

Lung abscess treatment results the treatment results improved with the development of antibiotics; however, surgical treatment is indicated when pyothorax is present, surgical treatment is indicated. When a lung abscess ruptures, pyothorax and fistula occur, which are difficult to treat.

**Case presentation:**

A 74-year-old woman who experienced exacerbated dyspnea and left back pain for 10 days was diagnosed with a lung abscess caused by an odontogenic infection. The patient’s medical history included hypertension, angina pectoris, untreated dental caries, and periodontitis. Despite administration of meropenem for 5 days, inflammatory markers increased. Chest radiography revealed pleural effusion exacerbation; therefore, the patient immediately underwent chest drainage and surgery was planned. Thoracic debridement and parietal and visceral decortication were performed. However, the lung abscess in the lateral basal segment ruptured during visceral decortication. As the tissue was fragile and difficult to close with sutures, free pericardial fat was implanted in the ruptured abscess cavity and fixed with fibrin glue, and sutured to the abscess wall. No signs of postoperative air leakage or infection of the implanted pericardial fat were observed. All drainage tubes were removed by postoperative day 9. The patient was discharged on postoperative day 12 and underwent careful observation during follow-up as an outpatient. At 1 year and 2 months after surgery, empyema recurrence was not observed.

**Conclusions:**

A lung abscess that ruptured intraoperatively was successfully and effectively treated by implantation of free pericardial fat in the abscess cavity.

## Background

At the beginning of the twentieth century, lung abscess (LA) was severely life-threatening disease, with a mortality rate of 75% [1]. However, treatment results for LA have improved with the development of antibiotics. Although antibiotic therapy has decreased the mortality rate associated with LA, it is still high (approximately 8.7%) [2]. Surgery is indicated for approximately 10% of LA cases, owing to complications such as bronchopleural fistula, empyema, and hemorrhage [3]. However, pyothorax and fistula occur upon rupturing of the LA, and are difficult to treat. We report the successful outcome of surgical treatment comprising free pericardial fat (FPF) implantation in the abscess cavity of an LA that ruptured intraoperatively.

## Case presentation

**Fig. 1:**
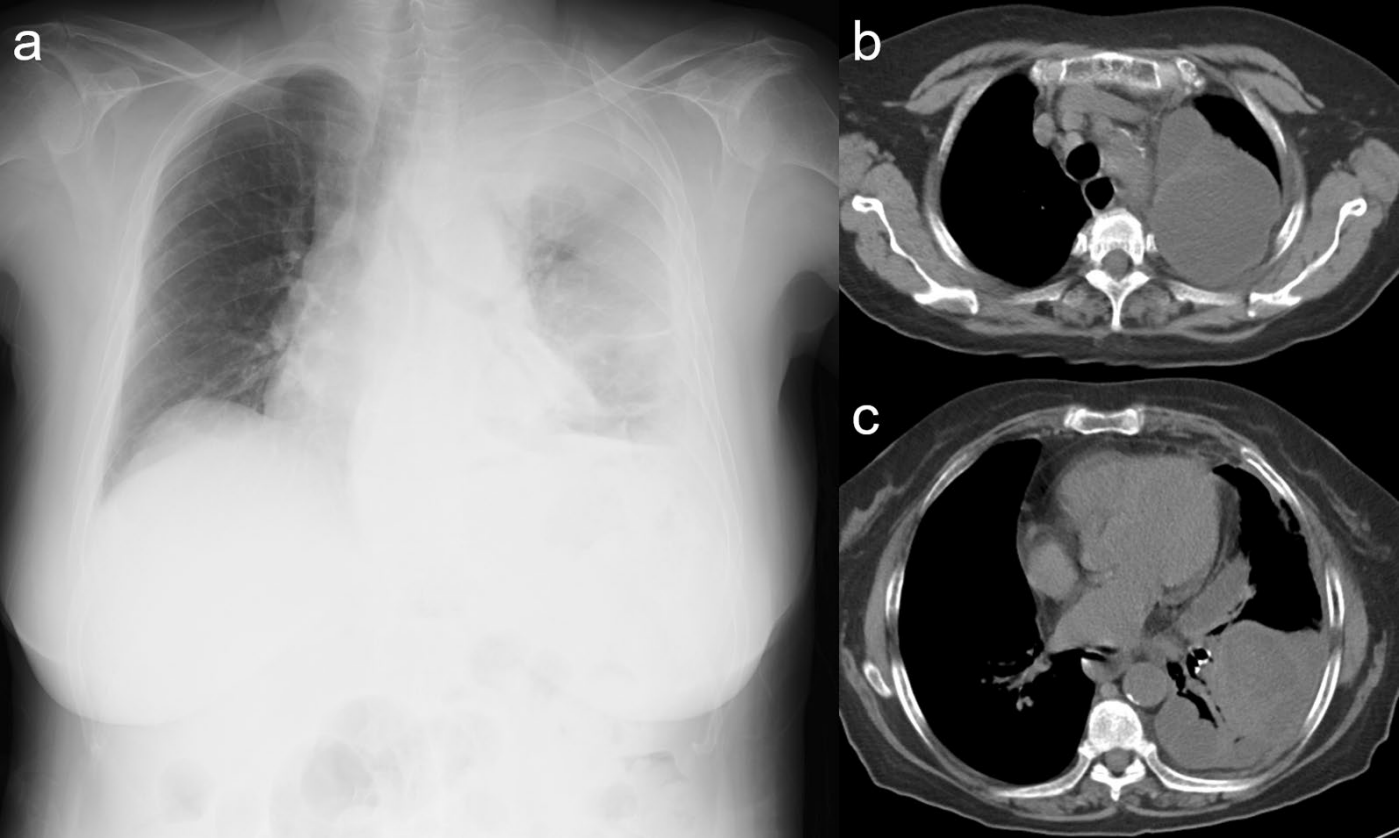
Chest radiography and computed tomography (CT) during the first visit. **a** Chest radiograph revealing collapse of the left lung and pleural effusion. Chest CT image showing, **b** multiple cavitated left pleural effusions and (c) a lung abscess in the left lower lobe

**Fig. 2:**
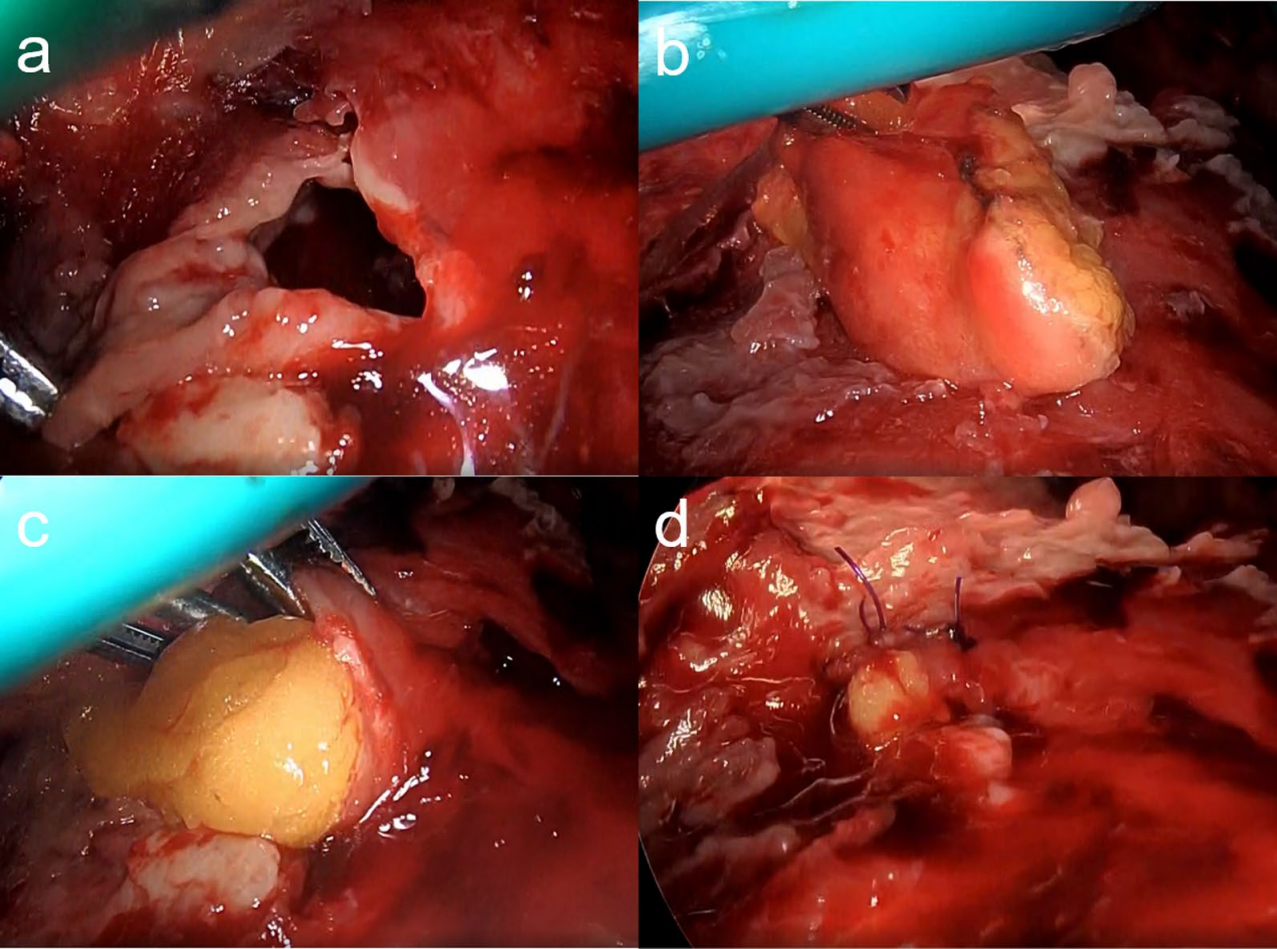
Intraoperative findings. **a** The abscess wall was fragile and torn during decortication. **b**, **c** Free pericardial fat (FPF) was implanted in the ruptured abscess cavity. (d) Fibrin glue was injected in the abscess cavity and FPF was sutured to the abscess wall

**Fig. 3:**
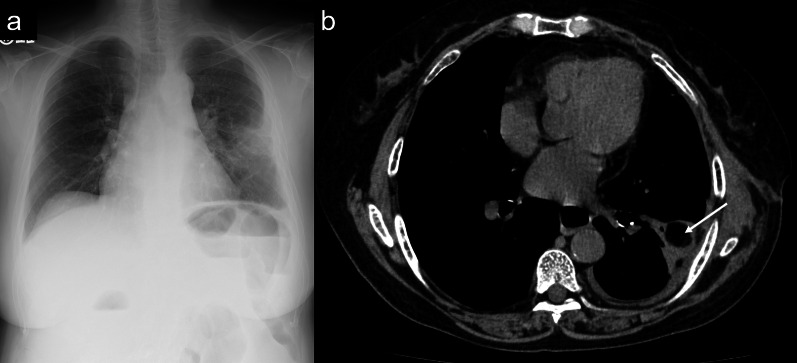
Chest radiography and computed tomography (CT) after discharge. **a** Chest radiograph showing decreased transparency in the middle lung field because of the implanted free pericardial fat (FPF); however, other parts of the lungs were well expanded. **b** Chest CT image showing the implanted FPF within the abscess cavity as a low-density area (white arrow)

A 74-year-old woman who experienced dyspnea exacerbation and left back pain for 10 days was diagnosed with an LA caused by odontogenic infection. Her medical history revealed comorbidities, such as hypertension, angina pectoris, untreated dental caries, and periodontitis. Although the patient was administered meropenem (MPMN) for 5 days, her body temperature reached 37.8 °C and inflammatory marker levels increased. Chest radiography revealed pleural effusion exacerbation (Fig. 1a). The patient’s white blood cell count and C-reactive protein level markedly increased to 21,130/µL and 33.74 mg/dL, respectively, and her procalcitonin level was elevated (0.28 ng/mL). Chest computed tomography (CT) revealed a large, multilocular pleural effusion and an LA in the left lower lobe (Fig. 1b, c). Although the patient immediately underwent chest drainage, poor drainage was observed in the apex. The pH of the pleural effusion markedly decreased to 6.9, and surgery was planned accordingly. First, thoracoscopic debridement was attempted; however, both the parietal and visceral pleura were markedly thickened. Therefore, we converted to thoracotomy, and performed parietal and visceral decortication. An LA was also observed in the lateral basal segment. The cause of empyema was perforation of the LA, and the abscess wall was fragile and torn during decortication (Fig. 2a). Large amounts of pus and air leakage were observed in the ruptured abscess cavity. Because the tissue was fragile, it was difficult to close with sutures. Therefore, FPF was implanted in the ruptured abscess cavity (Fig. 2b, c), fixed with fibrin glue, and sutured to the abscess wall (Fig. 2d). The thoracic cavity was irrigated with 10,000 mL of saline using a pulse lavage irrigation system. Air leakage was not observed with the 20cmH_2_O during the intraoperative leak test. Therefore, we considered that the abscess was sufficiently reduced, and that filling of the omental flap and reinforcement of the muscle flap were not necessary. Three drainage tubes were placed over the ventral lung apex, dorsal lung apex, and diaphragm. The operative time was 151 min, and the blood loss volume was 300 mL. Immediately after surgery, air leakage was not observed. On postoperative day (POD) 1, type 1 respiratory failure did not improve; therefore, bronchial sputum toileting was performed. Chest CT showed poor drainage on the mediastinal side; therefore, the patient underwent fibrinolytic therapy with intrathoracic urokinase to promote lung expansion on PODs 2 and 5. The culture detected *Streptococcus intermedius* was detected in the intraoperative pleural fluid and pus in the LA. Therefore, *Streptococcus intermedius* was the causative bacterium, and antibiotics treatment was de-escalated from MPEM to sulbactam/ampicillin on POD 6. Signs of postoperative air leakage and infection of the implanted FPF were not observed (Fig. 3a, b). All drainage tubes were removed by POD 9. The patient was discharged on POD 12 and underwent careful observation as an outpatient. At 1 year and 2 months after surgery, empyema recurrence was not observed.

## Discussion and conclusions

Despite the development of antibiotics and medical therapy, LA is a life-threatening disease with a high mortality rate [2]. Surgical interventions are indicated for cases refractory to medical therapy, life-threatening hemoptysis, cavitary lesions with a diameter larger than 6 cm, bronchopleural fistulas, prolonged sepsis and febricity, abscess rupture with pyopneumothorax, and empyema [2–4]. If surgical intervention is required, the risk of a bronchopleural fistula is increased [4].

The treatment of a ruptured or perforated LA is difficult. Including our case, 18 cases involving empyema associated with LA rupture or perforation have been reported (Table 1) [4–20]. The coexistence of diabetes mellitus occurred in eight cases [6–8, 10–12, 14, 19]. *Streptococcus* was identified in five cases [7, 9, 12, 14, 18]. Four patients who experienced respiratory failure or acute respiratory distress syndrome [9, 12, 13, 17], and two patients who experienced preoperative septic shock [4, 11] required mechanical ventilation and extracorporeal membrane oxygenation. Fenestration was performed during the first surgery for two cases [14, 19]. However, bronchial embolization using an endobronchial Watanabe spigot resulted in the successful in three cases [8, 12, 20]. Lobectomy was performed for three cases [4, 5, 13]. An omental or muscle flap was applied to the fistula in four cases [6, 7, 10, 19]. The fistula was directly sutured in three cases [7, 16, 17]. The abscess wall of our patient was severely damaged, and air leakage was difficult to control. Therefore, we implanted FPF in the ruptured abscess cavity because the abscess wall tissue was too fragile to close with direct suture. Subsequently, the abscess wall was sutured to the FPF.

**Table 1:** Reported cases of empyema associated with lung abscess rupture or perfolation

Case	Age	Sex	Site of LA	Comorbidities	Culture	Approach	Decortication	Debridement	Treatment for fistula	References
1	3	M	RML	–	Negative	Thoracotomy	+	ND	Rt middle lobectomy	[5]
2	18	M	LLL	Smith-Magenis syndrome, DM	Negative	Thoracotomy	+	ND	omental flap	[6]
3	40	M	RLL, LLL	DM	Rt: *Streptococcal sp.* Lt: Negative	VATS	+	ND	Rt S9: direct suture Rt S10: EWS → pedicled intercostal muscle flap Lt S10: free intercostal muscle flap	[7]
4	48	M	LUL	HT, DM	ND	Bronchoscopy	-	-	EWS, fibrin glue	[8]
5	51	M	LUL	ankylosing spondylitis, ARDS	*Streptococcus constellatus*	VATS	ND	ND	wedge resection	[9]
6	57	M	LLL	DM, RA	*Mycobacterium avium*	Thoracotomy	–	+	pedicled intercostal muscle flap	[10]
7	57	M	RML	septic shock	*Prevotella sp.*	Thoracotomy	–	+	EWS	[11]
8	58	M	RUL	RF, left pneumonia due to the inhalation of pus	*Streptococcus intermedius,* *prevotella buccae*	Bronchoscopy	–	–	EWS	[12]
9	60	M	RLL	asthma, arrythmia, RF	*Prevotella loescheii*	Thoracotomy	ND	ND	Rt middle and lower lobectomy	[13]
10	62	M	RML	DM	*Streptococcus Intermedius*	Fenestration	–	+	EWS → VAC	[14]
11	63	M	LLL	liver cirrhosis, DM	*Peptococcus sp.,* *Eubacterium sp*	VATS	+	+	EWS	[15]
12	63	M	RML	-	Negative	VATS	–	+	direct suture, PGA sheet, fibrin glue	[16]
13	20	F	RUL	septic shock, pulseless VT, MOF, DM, pulmonary tuberculosis	*Mycobacterium tuberculosis*	VATS	+	ND	Rt upper lobectomy	[4]
14	30	F	LLL	asthma, ARDS	Negative	VATS	+	ND	direct suture	[17]
15	55	F	RUL	HT	*Streptococcus angionosus group*	Thoracotomy	+	ND	ND	[18]
16	75	F	RUL	DM	*MRSA*	Fenestration	–	+	EWS → pedicled omental and muscle flap, thoracoplasty	[19]
17	87	F	RLL	microscopic polyangiitis	*Pseudomonas aeruginosa*	Bronchoscopy	–	–	EWS	[20]
18	74	F	LLL	HT, AP, dental caries, periodonitis	*Streptococcus intermedius*	VATS → Thoracotomy	+	–	FPF inplantation, direct suture, fibrin glue	Our case

AP: angina pectoris, ARDS: acute respiratory distress syndrome, DM: diabetes mellitus, EWS: endobronchial Watanabe spigot, F: female, FPF: free pericardial fat, HT: hypertension, LA: lung abscess, LLL: left lower lobe, Lt: left, LUL: left upper lobe, M: male, MOF: murtiple orgean failure, ND: not described, PGA: polyglycolic acid, RA: rhumatoid arthritis, RF: respiratory failuer, RML: right middle lobe, RLL: right lower lobe, Rt: right, RUL: right upper lobe, VAC: vacuum-assisted closure, VATS: video assisted thoracic surgery, VT: ventricular tachycardia

Coverage can be performed by suturing the use of FPF pad without artificial materials, resulting in the effective control of air leakage [21, 22]. In our department, FPF is actively used during surgery to cover bronchial stump, thus preventing bronchopleural fistulas [21]. Depending on the patient's body type, it is easy to handle and collect sufficient amounts of FPF. Although the abscess cavity was relatively large in our patient, we were able to collect a sufficient amount of FPF; therefore, the cavity was filled using FPF and fibrin glue. Furthermore, the fistula was controlled by suturing the FPF and fragile abscess wall. This is the first case report of the use of FPF for a ruptured LA.

In conclusion, we successfully treated an LA that ruptured intraoperatively. Therefore, FPF implantation in the ruptured abscess cavity can effectively treat this condition.

## Data Availability

All data generated or analyzed during this study are included in this published article.

## Abbreviations

LA: Lung abscess
FPF: Free pericardial fat
MEPM: Meropenem
CT: Computed tomography
POD: Postoperative day
